# Rush College

**Published:** 1883-04

**Authors:** 


### College Commencement Exercises.

ARTICLE IX.

RUSH COLLEGE.

The annual commencement exercises, of the forty-first year of this institution, were held in Central Music Hall on the 20th of February, at 2 o'clock P. M.

President Allen, in introducing the exercises of the day, referred to the fact that Rush College, having been chartered by the legislature 1837, was older than any other educational institution in Illinois. This was the close of its fortieth year of college work, and the occasion was signalized by the executive Faculty appearing in the traditional scholars robes of the ancient University of London, which had been adopted as the garb and insignia in which the Faculty would appear on all public occasions of the college.

Rev. Clinton Locke, D.D., offered prayer.

The president conferred the degree of *Doctor of Medicine* on one hundred and seventy-nine (179) gentlemen of the graduating class.

Dr. E. H. Van Patten, of the class, delivered a brief and very able valedictory.

Prof. Moses Gunn, M.D., LL.D., delivered the Doctorate address. (This address is printed in full in this number of the JOURNAL AND EXAMINER, and will be commented on next month.)

A large and cultivated audience was present, including many visiting alumni and many representatives of the local profession.

In the evening, the Faculty entertained the alumni of the college, and other invited guests, at a banquet at the Grand Pacific

Hotel. Over three hundred and fifty partook of the pleasures of an elaborate and comforting menu and of the still more enjoyable feast that followed it.

Prof. H. M. Lyman presided, and grace was said by Rev. T. E. Green.

The toast, "*Our Guests*," was responded to by Prof. J. N. Hyde, who uttered in eloquent words the welcome of the Faculty to the assembled company.

"*The Specialist*," was responded to by Dr. Horace Wardner, superintendent of the Insane Hospital at Anna, in a well-studied effort, which he read from manuscript. It was a masterful exposition of the true relations of the medical specialist, and was most heartily enjoyed by the company.

Gardner G. Willard, Esq., spoke "*As the Lawyers Look at Us*," in a finished and entertaining speech. We never knew before how much the two professions had in common, nor how great a bond of sympathy ought to exist between them.

Rev. Thos. E. Green spoke of "*Our Common Theme*" in a manner so eloquent, and in every way so well, that the company was fairly electrified by the effort.

A solid and much appreciated response to the toast, "*The Consultation*," was made by Dr. S. H. Birney, of Chanpaign.

Prof. J. Suydam Knox made a response to the sentiment, "*Quacks and Quackery*," which, for humor, fairly entitles him to be regarded as the humorist of the profession. His speech did as much for the digestion of the dinner that had just been eaten as wine could have done, and it was enjoyed much more.

Dr. J. B. Hench, of the class of '83, spoke to the interrogative sentiment, "*What Next?*" in a six minute speech replete with good sense, and set in such chaste English, and delivered with such grace of quiet dignity, as to elicit the plaudits of all present.

President Allen made the last speech of the evening, the sentiment being "*Predicaments*." It was felicitous, as such efforts on his part always are, and was a fitting finale to a galaxy of the most excellent after-dinner speeches that it has been our fortune to hear, on a single occasion, in many years.

Not a little of the enjoyment of the occasion was due to some excellent music rendered by a student and two alumni of this college, Messrs. Snyder, Davis and Bradley. They rendered a humorous duet, as follows:

### A MODERN CONSULTATION.

MUSIC—"Merry Maiden and Tar."—*Pinafore*.

DOCTOR. You tell me you've an ill-defined sensation.

PATIENT. Oh wise and learned doctor that you are!

DOCTOR. Connected with excessive cerebration.

PATIENT. Oh wonderful diviner that you are!

BOTH. Oh excessive cerebration;
Oh ill-defined sensation;
Oh wonderful diviner that you are!

DOCTOR. You're the victim of a mild hallucination,

PATIENT. You astonish me with such prodigious lore!

DOCTOR. And you have a growing inco-ordination.

PATIENT. Well, really, have I any trifle more?

BOTH. Oh the mild hallucination!
The inco-ordination, the bright irradition,
Of such scientific lore!

DOCTOR. The cortex of your frontal convolutions
I will now proceed to thoroughly explore;
And at once I reach intelligent conclusions—
How sad! your days of happiness are o'er.

BOTH. Those frontal convolutions, intelligent conclusions.

PATIENT. Oh melancholy tidings!
That my happiness is o'er.

DOCTOR. You are suffering from the gradual invasion
Of a fatal and insinuating spore,
Whose inevitable, cruel termination,
Is that you will surely come to be a bore.

PATIENT. Oh, doctor! you alarm me!
How really can it harm me?
And can you not forearm me
Against this deadly spore?

DOCTOR. You will shortly feel your cranium expanding,
And great will be the size which it attains;
And your knowledge will be ever so commanding
That not a thing unknown to you remains;

In your ear you'll have a flea,
In you bonnet then a bee,
And the "big head" is the title of this malady.

DOCTOR. 'Tis the work of the bacillus cerebrosus.

PATIENT. Why, doctor! seems to me I've heard of that.

DOCTOR. And the way to antisepticize the creature
Is for you to wear a brick within your hat.

BOTH. The bacillus cerebrosus,
Bacillus cerebrosus,
This very night we'll put a brick within our hats!

—DR. E. P. DAVIS.

The Alumni Association of the college held its annual meeting the morning of the 20th.

The committee on Prize Essay reported that two essays had been presented, but that neither was worthy of the prize. The same prize was continued for another year. The Association voted a donation of fifty dollars to the sufferers by the recent disaster to the mine at Braidwood.

The officers elected for the ensuing year were as follows: President, Dr. John Guerin, of Chicago; first vice-president, Dr. T. P. Russell, of Oshkosh; second vice-president, Dr. J. W. Fisher, of Milwaukee; secretary and treasurer, Dr. F. A. Emmons, of Chicago; executive committee, Drs. Maynard, Davis, and Bevan, of Chicago; prize essay committee, Drs. C. E. Parkes, C. T. Fenn, and J. S. Knox.

